# Modulation of Microglial Activation by Adenosine A2a Receptor in Animal Models of Perinatal Brain Injury

**DOI:** 10.3389/fneur.2018.00605

**Published:** 2018-09-11

**Authors:** Marina Colella, Manuela Zinni, Julien Pansiot, Michela Cassanello, Jérôme Mairesse, Luca Ramenghi, Olivier Baud

**Affiliations:** ^1^Robert Debré Hospital, PROTECT, Inserm U1141, Paris, France; ^2^Istituto G. Gaslini, Università di Genova, Genoa, Italy; ^3^Laboratory for the Study of Inborn Errors of Metabolism, Istituto Giannina Gaslini, Genoa, Italy; ^4^Division of Neonatology and Pediatric Intensive Care, Children's University Hospital of Geneva, University of Geneva, Geneva, Switzerland; ^5^Neonatal Intensive Care Unit, Istituto Giannina Gaslini, Genoa, Italy

**Keywords:** adenosine, inflammation, brain damage, fetal growth restriction, prematurity, microglia

## Abstract

Neuroinflammation has a key role in the pathogenesis of perinatal brain injury. Caffeine, a nonspecific antagonist of adenosine receptors (ARs), is widely used to treat apnea of prematurity and has been linked to a decrease in the incidence of cerebral palsy in premature infants. The mechanisms explaining its neuroprotective effect have not yet been elucidated. The objective of this study was to characterize the expression of adenosine and ARs in two neonatal rat models of neuroinflammation and to determine the effect of A2aR blockade on microglial activation assessed through inflammatory cytokine gene expression. We have used two rat models of microglial activation: the gestational low protein diet (LPD) model, associated with chronic brain injury, and postnatal ibotenate intracerebral injections, responsible for acute excitotoxicity injury. Adenosine blood levels have been measured by Tandem Mass Spectrometry. The expression of ARs *in vivo* was assessed using qPCR and immunohistochemistry. *In vivo* models have been replicated *in vitro* on primary microglial cell cultures exposed to A2aR agonist CGS-21680 or antagonist SCH-58261. The effects of these treatments have been assessed on the M1/M2 cytokine expressions measured by RT-qPCR. LPD during pregnancy was associated with higher adenosine levels in pups at postnatal day 1 and 4. A2aR mRNA expression was significantly increased in both cortex and magnetically sorted microglial cells from LPD animals compared to controls. CD73 expression, responsible for extracellular production of brain adenosine, was significantly increased in LPD cortex and sorted microglia cells. Moreover, CD73 protein level was increased in ibotenate treated animals. *In vitro* experiments confirmed that LPD or control microglial cells exposed to ibotenate display an increased expression, at both protein and molecular levels, of A2aR and M1 markers (IL-1β, IL-6, iNOS, TNFα). This pro-inflammatory profile was significantly reduced by SCH-58261, which reduces M1 markers in both LPD and ibotenate-exposed cells, with no effect on control cells. In the same experimental conditions, a partial increased of M1 cytokines was observed in response to A2aR agonist CGS-21680. These results support the involvement of adenosine and particularly of its receptor A2aR in the regulation of microglia in two different animal models of neuroinflammation.

## Introduction

Brain injury is one of the most important complication related to preterm birth (1). From 25 to 50% preterm infants display neurodevelopmental disabilities (2), with dramatic consequences in terms of cost and impact on quality of life.

Preclinical and clinical studies show that neuroinflammation plays a central role in the pathogenesis of perinatal brain damage (3–8). One of the first events following neuroinflammation is activation of microglia cells (9), that assume different phenotypes, conventionally classified as M1 (pro-inflammatory) and M2 (anti-inflammatory, reparative) (10, 11).

During the inflammatory process, extracellular adenosine, an ubiquitous molecule implicated in neuromodulation, reaches high concentrations capable of activating the adenosine receptors (ARs), denoted A1, A2a, A2b, and A3 (12, 13).

The most implicated adenosine receptor in neuroinflammation is A2aR (14). Its expression in microglia is usually low but increases following brain insults. In microglial cells, activation of A2aRs has facilitating effects on the release of cytokines (15) and on the change into amoeboid morphology (16). Conversely, A2aR antagonists suppress microglia activation, as described using *in vitro* (17, 18) and *in vivo* (18) studies.

To our knowledge, there are no data regarding the adenosine pathway and neuroinflammation in preterm infants, but nevertheless, the involvement of adenosine signaling in prematurity is suggested by the clinical use of caffeine. Indeed, caffeine, a non-specific antagonist of ARs widely used to treat apnea of prematurity, not only improves survival and reduces the duration of respiratory support, but also reduces the incidence of cerebral palsy and cognitive delay (19). Recently, a retrospective study demonstrated the existence of high blood levels of adenosine in premature infants (20), with the highest adenosine concentrations associated with the lowest birthweight.

These data suggest a possible link between caffeine action, adenosine plasma levels and an imbalance between the pro- and anti-inflammatory profiles in very preterm infants usually delivered following a perinatal inflammatory event. Whether a similar link exists for adverse neurological outcomes in preterm infants is not known and there is still little evidence relating to effects of caffeine on brain development, especially at the cellular and molecular levels (21).

Therefore, this study was aimed to characterize the synthesis and expression of adenosine and its receptors in two experimental animal models of neonatal neuroinflammation. The effect of A2aR blockade was also studied *in vitro* using a specific antagonist on microglial activation assessed through pro- and anti-inflammatory cytokine gene expressions.

## Materials and methods

### Animals and models

All experiments were carried out according to INSERM ethical rules and approved by the institutional review board (Robert Debré ethics committee, Paris, France, approval number Big Project 01542.01). Sprague-Dawley rats (Janvier SAS, Le Genest-St-Isle, France) were housed in temperature-controlled rooms (24°C), with 12 h light cycling and free access to chow and water *ad libitum*.

#### Low protein diet (LPD) model

After mating, dams were randomly allocated to either isocaloric low-protein diet (LPD) (9% casein; as previously described (22, 23), SAFE-diets Augy, France) or control diet (CTL) (23% casein) during the gestational period. The control and LP diets are balanced for energy intake assuming equivalent consumption rates. At birth, dams were returned to standard diet. Sex, birthweight and postnatal growth rates were determined. Experiments research plan using this model is summarized in Supplemental Figure 1.

#### Ibotenate (IBO) model

Ten μg IBO diluted in Phosphate Buffered Saline (PBS) was injected intracerebrally (i.c.) at postnatal day 5 (P5) to rat pups of both sexes as previously described (24). Experiments research plan using this model is summarized in Supplemental Figure 2.

The rat pups were killed and dissected at different postnatal days (P1, P4, and P5). Blood was collected by exsanguination on filter paper. Brains were collected, immediately snap frozen and stored at −80°C or immediately dissociated for microglia cells isolation.

### Antibodies and reagents

Ibotenate (IBO, Tocris, Bristol, UK) was diluted in PBS to prepare a stock solution of 20 mg/ml. SCH-58261 (SCH; Sigma Aldrich, Lyon, France S4568) and CGS-21680 hydrochloride hydrate (CGS; Sigma Aldrich, Lyon, France, C141) were diluted in DMSO to a stock concentration of 10 mM. Primary antibodies: anti-A2aR antibody (rabbit polyclonal, ab3461); anti-CD73 antibody (rabbit polyclonal, ab175396); anti-ionized calcium-binding adaptor protein-1 antibody (anti-Iba1, goat polyclonal, ab5076) were all purchased from Abcam (France). Goat anti-rabbit and anti-mouse IgG conjugated to horseradish peroxidase were purchased from Sigma (Lyon, France). AlexaFluor® 488-conjugated anti-goat IgG and DAPI were from Life Technologies, while Cy3-conjugated anti-rabbit IgG was from Jackson Immuno Research Laboratories.

### Microglia cell isolation and primary culture

Brains were collected from control and LPD animals at P1 and P4 removing the cerebellum and the olfactory bulbs. The tissues dissociation was performed using the Neural Tissue Dissociation Kit and the gentleMACS Octo-Dissociator with Heaters accordingly to the manufacturer's instruction (Miltenyi Biotec, Germany). CD11b positive cells were isolated from the resulting homogenates using an anti-CD11b (microglia marker) MicroBeads (Miltenyi Biotec, Germany) and multiMACSCell-24 separator (Miltenyi Biotec, Germany). After elution the sorted microglia cells were stored at −80 for RNA extraction. In a second set of experiments, microglia cells were magnetically sorted from control and LPD animals at P4 and after elution pellet was isolated by centrifugation (300 g - 10 min). Following re-suspension in Dulbecco's modified Eagle's minimum essential medium/Nutrient mixture F-12 (DMEM/F-12, Gibco) supplemented with 10% fetal bovine serum (FBS, Gibco) and 1% penicillin/streptomycin (P/S), cells were maintained in DMEM/F-12 supplemented with 10% FBS and 1% P/S at a concentration of 5 × 10^5^ cells/ml in 12-well culture plates. The purity of isolated microglia cells was verified by Iba1 immunostaining (dilution 1/1,000). A medium change was performed after 24 h and cells were treated as follows after 48 h. Microglial cells were treated with SCH-58261 (A2aR antagonist) at 50 nM (17, 25) or CGS-21680 (A2aR agonist) at 10 μM (26, 27) or DMSO. For the IBO model, cells sorted at P4 were treated with SCH, CGS or DMSO 20 min before adding ibotenate 300 μM (28). After 6 h, cells were harvested and RNA extracted for gene expression analysis. For cytokine levels, supernatant (conditioned media) was collected after a longer exposure time (12 h) and stored at −80°C until analysis.

### RNA extraction, retro-transcription and real-time PCR

Total RNA was extracted from cortex using Qiazol reagent and RNeasy mini kit (Qiagen, France) and from microglia cells using the NucleoSpin RNA Plus extraction kit (Macherey-Nagel, France) according to the manufacturer's instructions. RNA yield and purity were determined by spectrophotometry absorption at 260 and 280 nm by means of a NanodropTM apparatus (Thermofisher Scientific, MA, USA). Five hundred ng of mRNA from cortex and 150 ng from microglia were used to perform reverse transcription (iScript TM cDNA synthesis kit, Biorad, France), respectively. qPCR measurements were performed in duplicate using SYBR Green Super-mix (Bio-Rad). The reaction conditions were as follows: 98°C for 10 min (Polymerase activation), followed by 45 cycles at 95°C for 5 min, 60°C for 10 min and 72°C for 10 min. The specificity of used primers was assessed with a melting curve analysis and the results were quantified using the relative standard curve methods. The relative mRNA expression for each target gene was calculated after normalization respect to the Rpl13 references gene. The primers sequences are available in Supplemental Table 1.

### Multiplex cytokine assay

Cytokines were measured using the Bio-Plex rat cytokine multiplex kit (Bio-Rad). Calibration curves from recombinant cytokine standards were prepared with serial dilutions in the same media as the culture supernatant (DMEM/F-12 supplemented with 10% FBS and 1% P/S). Standards and samples were analyzed in duplicate and blank values were subtracted from all readings. All assays were carried out directly in a 96-well filtration plate (Bio-Rad) at room temperature and protected from light. Briefly, wells were pre-wetted with culture supernatant, then beads together with either standard, sample, or blank were added in a final volume of 50 μl, and incubated together at room temperature for 30 min with continuous shaking. Beads were washed three times with 100 μl Bio-Plex wash buffer. A cocktail of biotinylated antibodies (25 μl/well) was added to the beads for a further 30-min incubation with continuous shaking. Beads were washed three times, then streptavidin-phycoerythrin was added for 10 min. Beads were again washed three times and resuspended in 125 μl assay buffer. The fluorescence intensity of the beads was measured using the Bio-Plex array reader. Bio-plex manager software with five-parametric-curve fitting was used for data analysis.

### Immunofluorescence assay and quantification

For histological analysis after ibotenate i.c., injections, animals were anesthetized with pentobarbital and transcardially perfused with 4% paraformaldehyde in PBS. Brains were collected, postfixed in 4% paraformaldehyde overnight, cryoprotected, cut coronally in 10 μm-thick slices, and stained according to standard protocols. After three washings of the slices with PBS, the non-specific binding was blocked by incubating the tissue sections with PBS-Triton 0.5%-gelatin 0.2% for 45 min at room temperature. Incubation with primary antibodies (rabbit anti-CD73 1/1,000; goat anti-Iba1 1/1,000) was performed overnight at 4°C in PBS-Triton 0.5%-gelatin 0.2%. After rinsing three times in PBS for 5 min each, sections were exposed (1 h, room temperature) to secondary species-specific antibodies (all at 1/1,000 dilution in PBS-Triton 0.5%-gelatin 0.2%) conjugated to Alexa Fluor® 488 or to Cy3. Nuclei were then labeled with the fluorescent DAPI dye (1/10,000 in PBS). Stained sections were mounted on microscope slides with Fluoromount-G (SouthernBiotech).

Primary microglia cells cultured in micro-slide 8-well chamber (Ibidi, Germany) and treated as reported above were fixed in 4% paraformaldehyde for 30 min at room temperature. Each well was incubated with a blocking solution (PBS with 1% BSA) for 1 h at room temperature and incubated overnight at 4°C with goat anti-Iba1 (1/500) and anti-A2aAR (1/250). The following day, after rinsing three times in PBS for 5 min, cells were incubated with secondary antibodies coupled to the green and red fluorescence markers (1/500 dilution) for 1 h at room temperature. Nuclei were visualized by staining the cells with DAPI dye (1/10,000).

Cells were analyzed using a fluorescent microscope (Nikon Eclipse Ti-E) and images captured with a 20X objective (4 wells/group and 5 images/well). Fields used for quantitation were randomly selected throughout the dish and focused using phase contrast optics. Images from different emission specters were acquired separately using the same parameters and superimposed in the aftermath. For the analysis Image J software (Research Service Branch, National Institutes of Health, Bethesda, MD; http://rsb.info.nih.gov/ij/) was used. Images were converted to binary images using an automatic threshold function. The cells in each image were then defined by outlining a mask image (ROI). For LPD experiments, as the cells showed a significant difference in cell size due to microglial activation, the fluorescence power was calculated as the mean of all the pixel intensities of each individual cell. For the ibotenate experiment, the fluorescence power was calculated as an integrated density (i.e., the product of the mean of all the pixel intensities of each individual cell and the ROI area). Cell size was calculated using Iba1-positive cells as the product of number of pixels in ROI and the conversion factor 0,103. Finally, the sums of the values for each condition were normalized to control values for the statistical analysis.

### Statistical analysis

The graphs and the statistical analysis were performed with GraphPad Prism 5.0 (GraphPad Software, San Diego, CA). Appropriate statistical analyses were carried out with a two-sided alpha level of 0.05 or 95% confidence interval. For continuous data, descriptive analyses were carried out employing means and ± SEM, Parametric or non-parametric tests were used to compare quantitative variables (Student's *t*-test for independent samples for comparisons between two groups and one-way ANOVA with Newman Keuls post-test for comparisons of more than two groups). A *p*-value < 0.05 was considered statistically significant.

## Results

### The LPD model influences adenosine blood levels and the cerebral expression of enzymes and receptors implicated in adenosine metabolism

**Figure 1:**
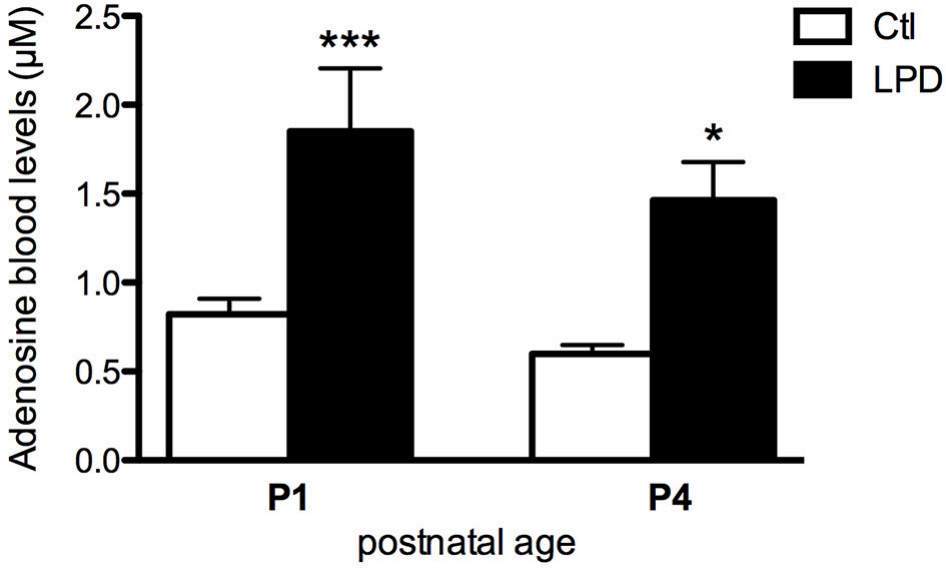
Adenosine blood levels in animals exposed to antenatal low protein diet (LPD) and in control (Ctl) animals at postnatal day P1 and P4. Data are shown as the mean value ± S.E.M. (^*^p < 0.05, ^***^p < 0.001, using unpaired Student's *t*-test).

Prenatal LPD-induced malnutrition resulted in the absence of postnatal mortality but fetal growth restriction with body weight of the pups significantly lower from P0 to at least P4 compared to the controls (Supplemental Figure 4). LPD pups at P1 and P4 showed significantly higher adenosine blood levels compared to the control pups (0.822 ± 0.088 μM in Controls vs. 1.850 ± 0.355 μM in LPD at P1, 0.598 ± 0.051 μM in Controls vs. 1.464 ± 0.215 μM LPD at P4, see Figure 1).

**Figure 2:**
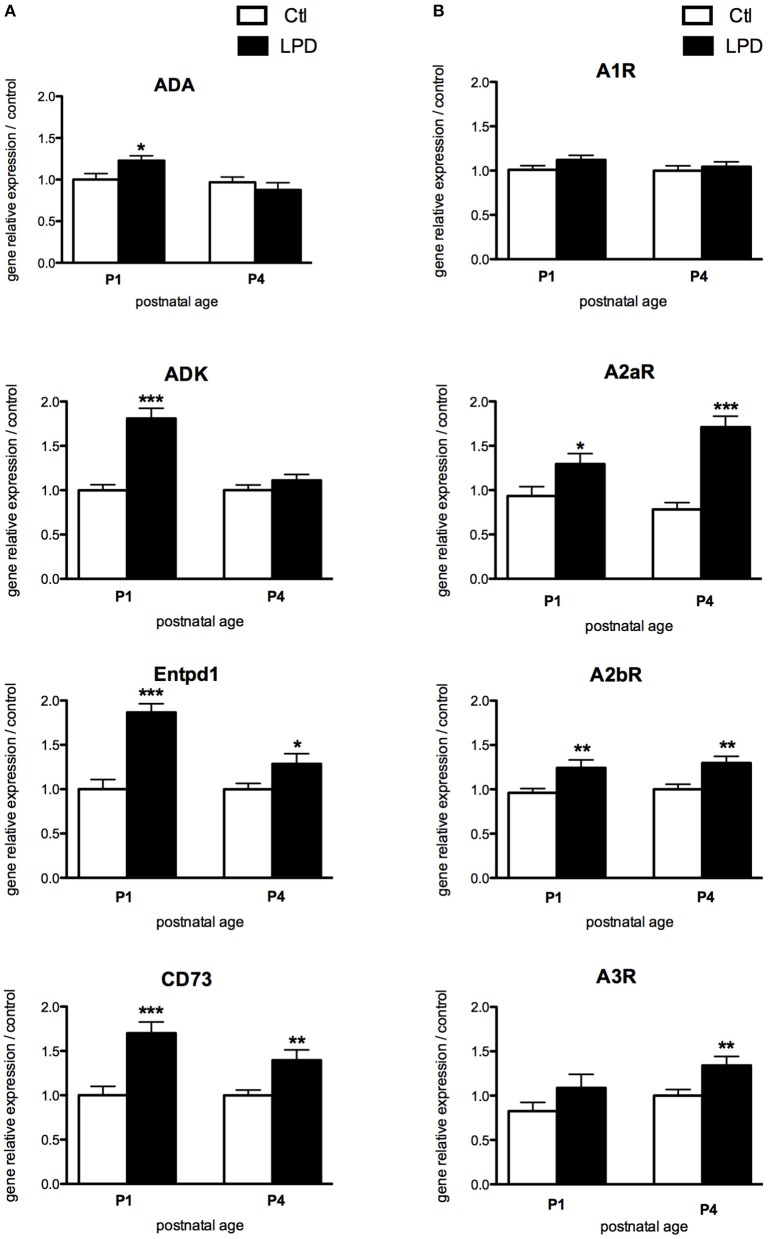
Comparisons of gene expression of enzymes involved in the production of adenosine **(A)** and its receptors **(B)** in animals exposed to LPD (black bars) and in control animals (white bars) in prefrontal cortex. Data are shown as relative expression of control values normalized to 1 (^*^p < 0.05, ^**^p < 0.01, ^***^p < 0.001, using unpaired Student's *t*-test). ADA, adenosine deaminase; ADK, adenosine kinase; Entpd1, ectonucleoside triphosphate diphosphohydrolade-1; CD73, cluster of differentiation 73; A1R, adenosine A1 receptor; A2aR, adenosine A2a receptor; A2bR, adenosine A2b receptor; A3R, adenosine A3 receptor.

In the pre-frontal cortex of LPD animals, the expression of adenosine deaminase (ADA), adenosine kinase (ADK), ectonucleoside triphosphate diphosphohydrolade-1 (Entpd1) and cluster of differentiation 73 (CD73), enzymes involved in the regulation of extracellular and intracellular adenosine levels, were significantly increased at P1, compared to controls (Figure 2A). The relative gene expression of intracellular enzymes ADA and ADK were comparable between LPD and controls at P4, whereas the two ectonucleotidases Entpd1 and CD73 are persistently increased at this age in the LPD group, indicating an increased extracellular production of adenosine. A2aR and A2bR, the two main ARs with pro-inflammatory functions, were significantly up-regulated at P1 and P4 in the cortex of LPD pups (Figure 2B), suggesting a pro-inflammatory status of the LPD brains. In contrast, no significant differences were observed at a transcriptional level for A1 and A3 receptors, except for an increased expression of A3R at P4 in the LPD group (Figure 2B).

### A2aR expression is increased in microglial cells sorted from the rat pups subjected to antenatal LPD

**Figure 3:**
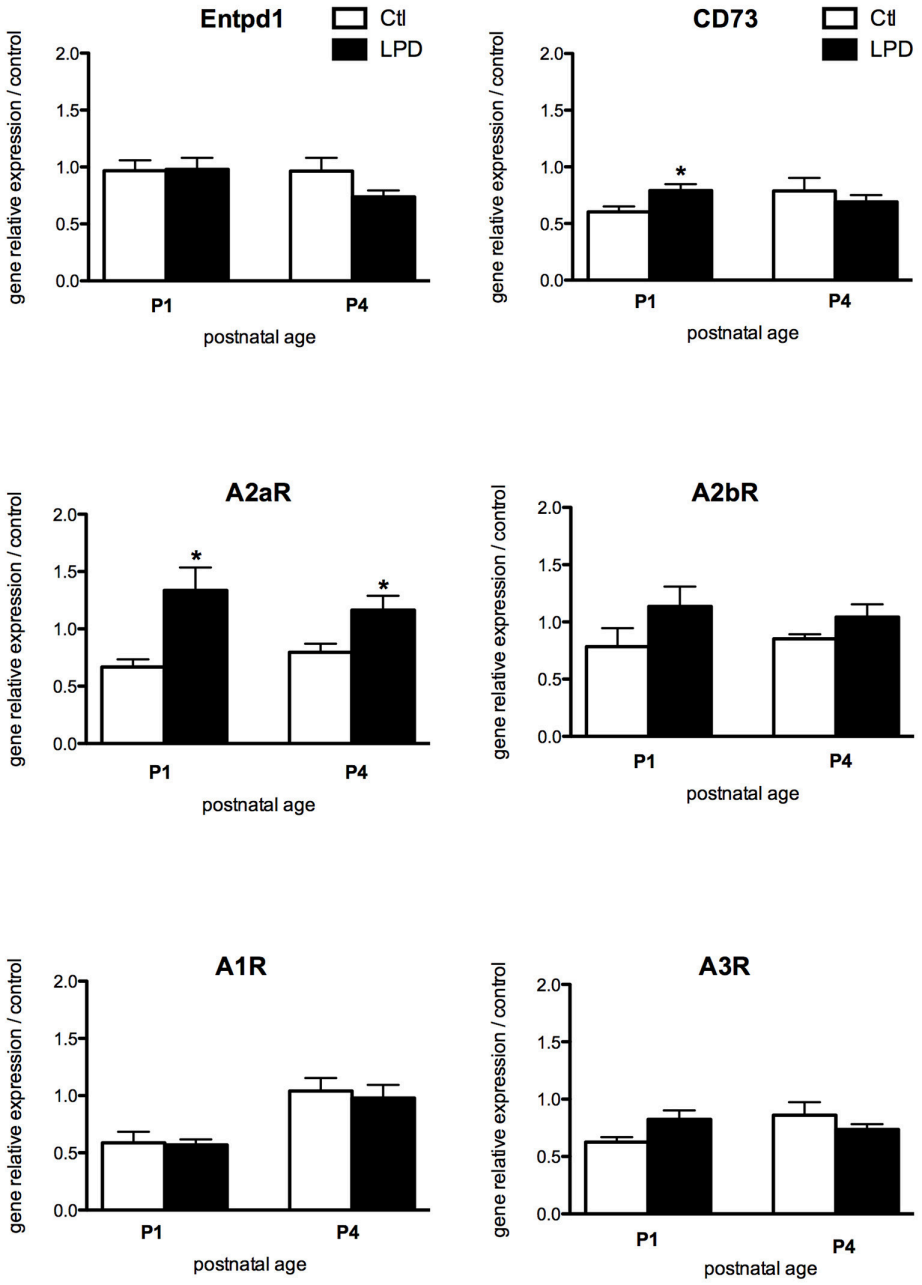
Comparisons of gene expression of enzymes involved in the production of adenosine and its receptors in microglial cells sorted from animals exposed to LPD (black bars) and from control animals (white bars). Data are shown as relative expression of control values normalized to 1 (^*^p < 0.05, using unpaired Student's *t*-test).

While most of the receptors display a similar pattern of expression, A2aR expression was found significantly increased in microglia cells sorted from LPD brains compared to controls, both at P1 and P4 (Figure 3). Finally, no statistically significant difference was observed in the expression of genes encoding for the enzymes involved in the adenosine extracellular metabolism, except for a transient and mild increase in the expression of CD73 at P1 in LPD animals.

**Figure 4:**
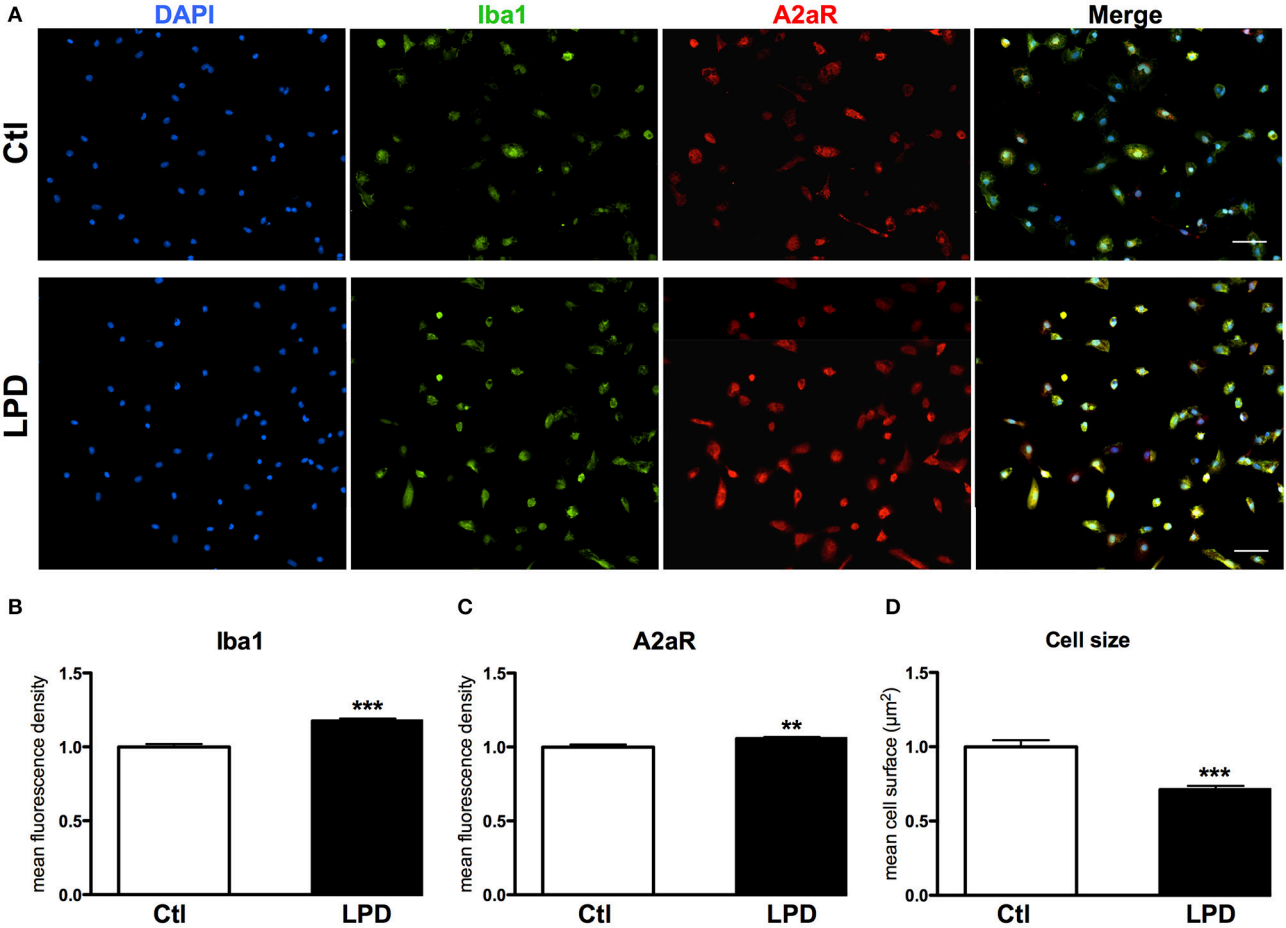
A2aR expression in microglial cells sorted from animals exposed to LPD. **(A)** A2aR (red) and Iba-1 (green) immunoreactivity microglial cells sorted from LPD-exposed rat pups and control animals (scale bar: 50 μm). **(B,C)** Quantification of mean fluorescence for A2aR and Iba-1 immunoreactivity in control and LPD microglial sorted cells. **(D)** Quantification of cell size on Iba-1 positive cells. Data are shown as relative expression of control values normalized to 1 (^**^p < 0.01, ^***^p < 0.001, using unpaired Student's *t*-test).

The mean density of A2aR immunoreactivity in microglial cells after 48 h in culture was significantly higher in LPD compared to control group (Figures 4A,B). Moreover, Iba1 immunoreactivity was found increased and cell size reduced in LPD microglial cells, compared to control cells (Figures 4C,D).

### A2aR antagonist exposure changes microglial reactivity *in vitro*

**Figure 5:**
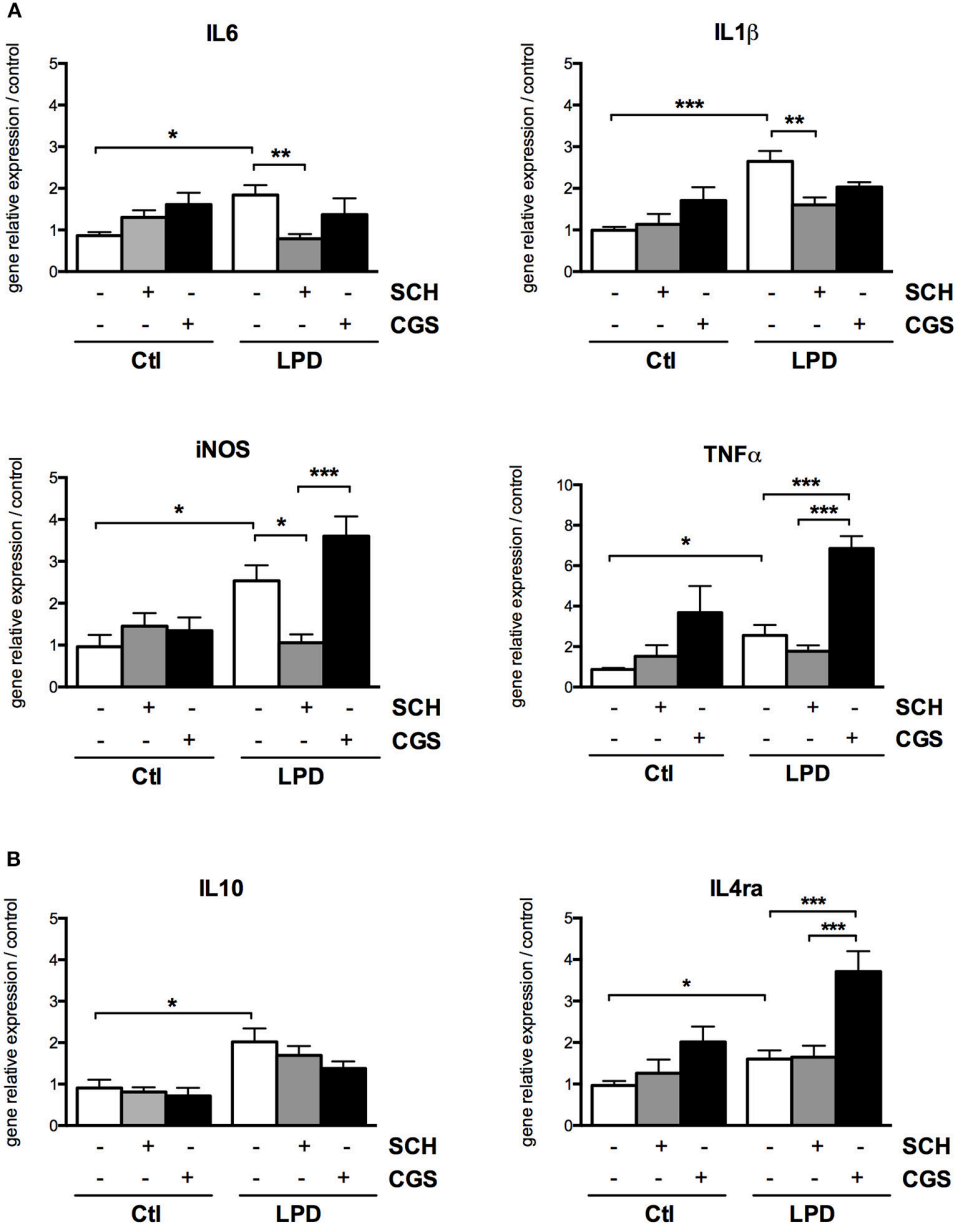
A2aR modulation regulates the phenotype of microglial cells sorted from LPD animals. Quantification of gene relative expressions of M1 **(A)** and M2 **(B)** proteins in microglial cells treated with or without SCH-58261 (A2aR antagonist) and CGS-21680 (A2aR agonist) sorted from control or LPD animals (^*^p < 0.05, ^**^p < 0.01, ^***^p < 0.001, using one-way ANOVA followed by Newman-Keuls *post-hoc* analysis).

After 2 day, significant increases in gene expression of both M1 (IL-1β, IL-6, iNOS, TNFα) and M2 markers (IL-10, IL-4ra) were detected in microglial cells sorted from LPD rat pups, compared to control cells (Figure 5). SCH-58261, an A2aR antagonist, induced a significant reduction in the expression of M1 markers while no effect on M2 markers was detected in LPD microglial cells. Conversely, the A2aR agonist CGS-21680 was able to increase the mRNA levels of iNOS, TNFα and IL-4ra in LPD microglial cells. No substantial effect of either SCH-58261 or CGS-21680 was observed in control cells.

### A2aR plays a role in excitotoxic-induced microglial activation

In the IBO model assessed 24 h after ibotenate injection, 1/3 of Iba-positive cells surrounded the white matter lesion expressed CD73, an enzyme responsible for the extracellular production of adenosine in the brain (Supplemental Figure 5).

**Figure 6:**
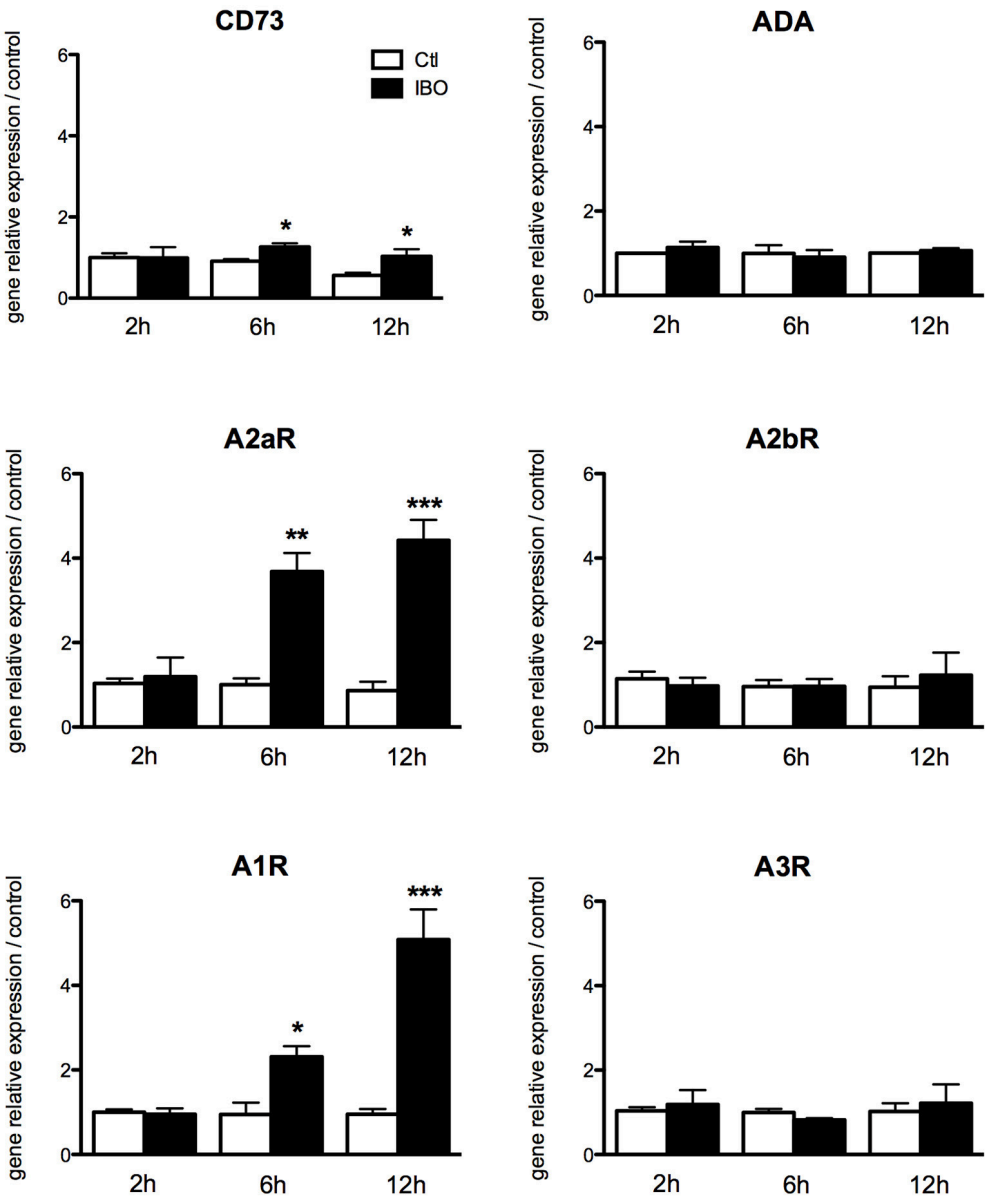
Effect of ibotenate exposure to microglial cell cultures on gene expression of enzymes involved in the production of adenosine and its receptors. Data are shown as relative expression of control values (PBS exposure) set to 1 for each time-point. (^*^p < 0.05, ^**^p < 0.01, ^***^p < 0.001, using unpaired Student's *t*-test). CD73, cluster of differentiation 73; A2aR, adenosine A2a receptor; A1R, adenosine A1 receptor; ADA, adenosine deaminase; A2bR, adenosine A2b receptor; A3R, adenosine A3 receptor.

To assess the putative involvement of the adenosine pathway in neuro-inflammation induced by excitotoxicity, magnetically sorted microglial cells from P4 control pups were exposed to 300 μM IBO for different lengths of time from 2 to 12 h. The exposure to IBO was unable to induce an increase in A1 and A2a receptor expression levels after 2-h exposure (Figure 6). In contrast, a significant and progressive increase in the gene expression of both receptors was observed after 6- and 12-h IBO exposure. CD73 was also found to be slightly but significantly increased, while no difference was observed regarding A2b and A3 receptors gene expression.

**Figure 7:**
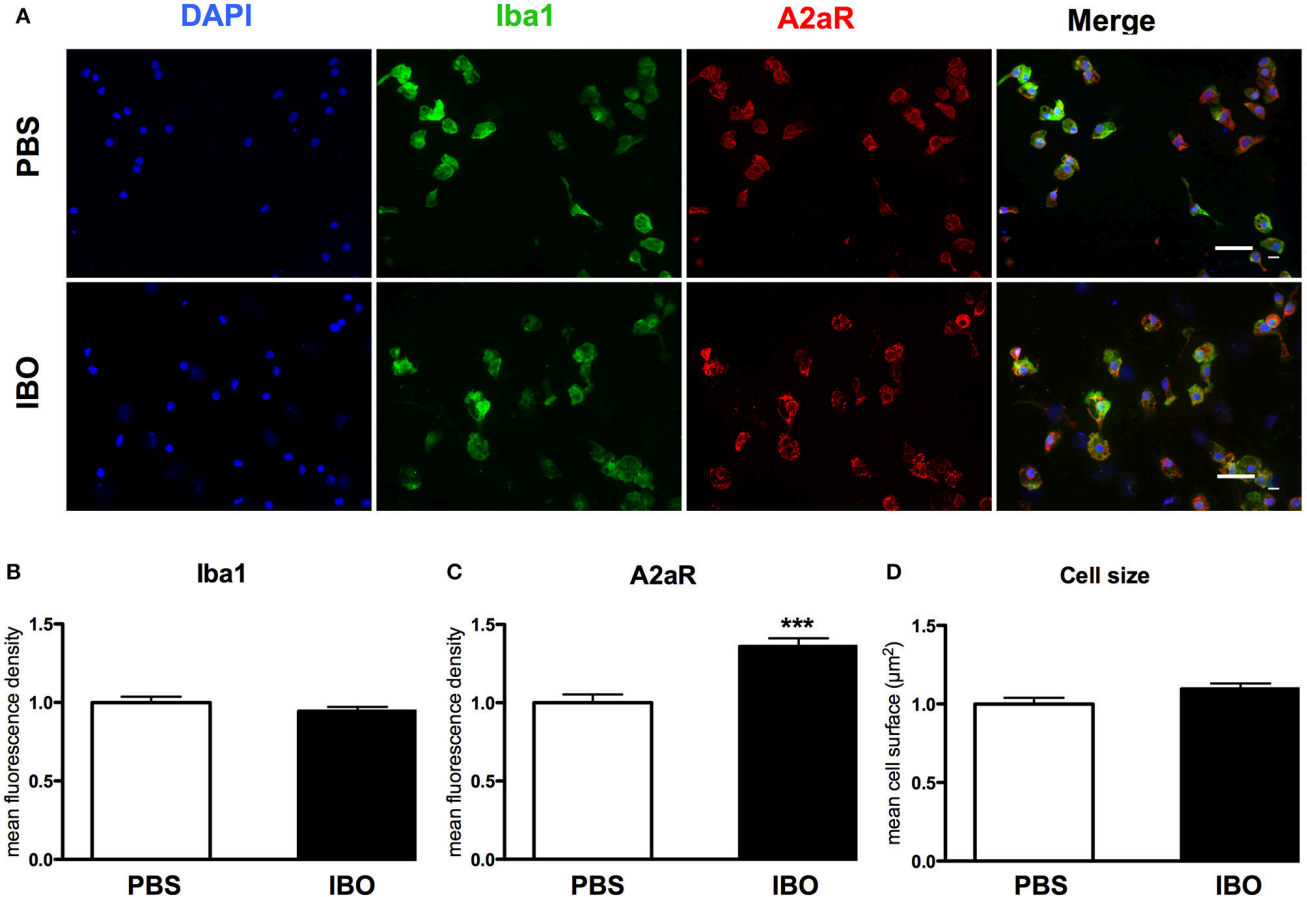
A2aR expression in sorted microglial cells exposed to ibotenate (IBO). **(A)** A2aR (red) and Iba-1 (green) immunoreactivity in microglial cells sorted from P4 rat pups after 6 h IBO or PBS exposure (scale bar: 50 μm). **(B,C)** Quantification of mean fluorescence for Iba-1 and A2aR immunoreactivity in PBS and IBO-exposed cells. **(D)** Quantification of cell size on Iba-1 positive cells. Data are shown as relative expression of PBS values normalized to 1 (^***^p < 0.001, using unpaired Student's *t*-test).

These results were confirmed at protein level using immunocytochemistry (Figure 7). IBO exposure for 6 h was associated with a significant increase in A2aR immunoreactivity in microglial cells, while cell size and the Iba1 immunoreactivity were found similar with and without IBO.

### A2aR modulation is involved in the regulation of M1-M2 microglia phenotype in excitotoxic-induced inflammation

**Figure 8:**
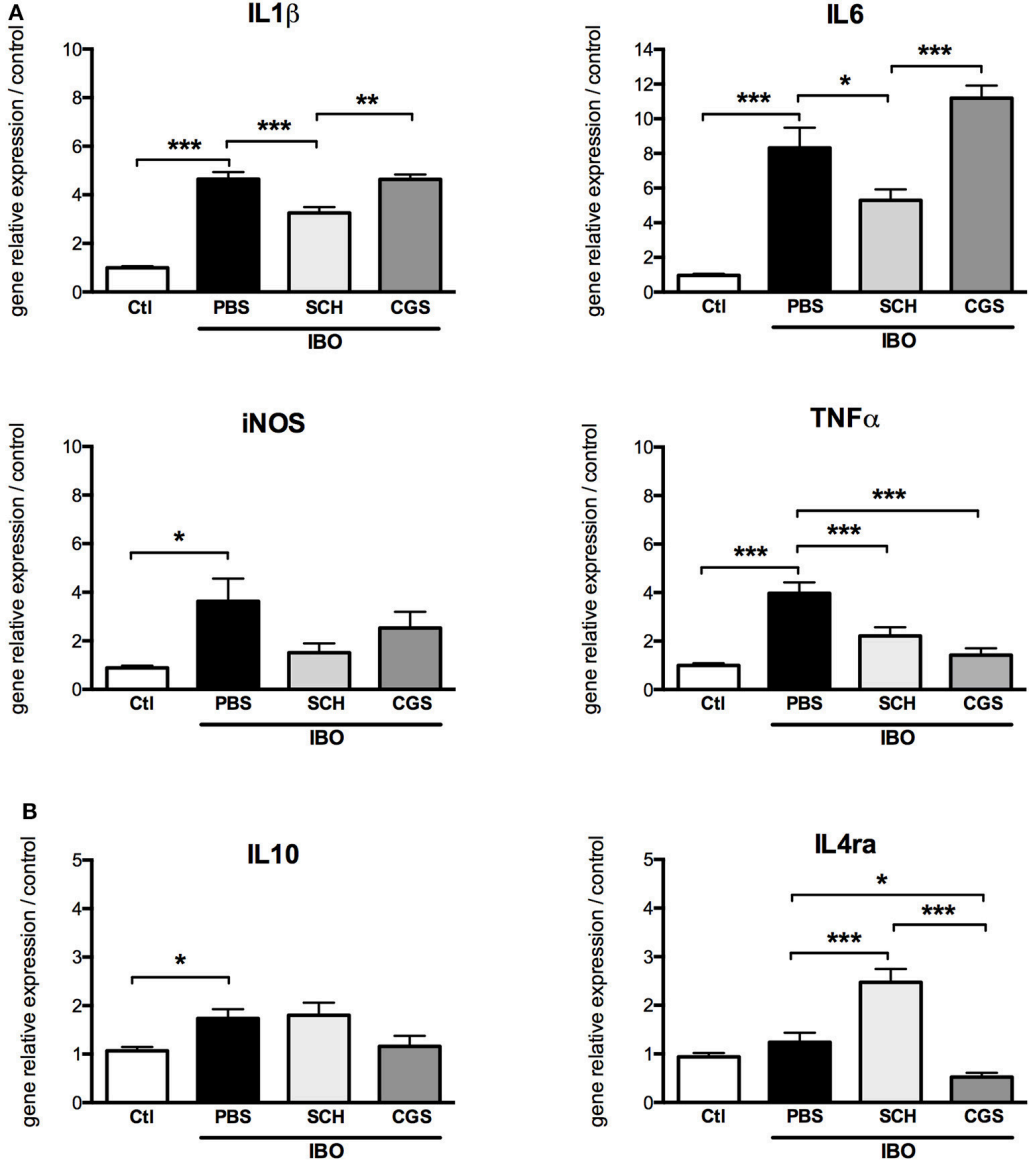
A2aR modulation regulates the phenotype of microglial sorted cells exposed to 300 μM ibotenate (IBO) for 6 h. Quantification of gene relative expressions of M1 **(A)** and M2 **(B)** proteins in microglial cells treated with or without SCH-58261 (A2aR antagonist) and CGS-21680 (A2aR agonist) sorted from control or LPD animals (^*^p < 0.05, ^**^p < 0.01, ^***^p < 0.001, using one-way ANOVA followed by Newman-Keuls *post-hoc* analysis).

In our *in vitro* model of excitotoxic activation of primary microglial cells, highly significant increase in gene expressions of IL1β, IL6, iNOS, TNFα, and IL10 were was observed in response to 300 μM IBO exposure for 6 h (Figure 8). This effect was significantly reduced by SCH-58261 pre-treatment, 20 min before IBO challenge. Interestingly, SCH-IBO treated microglia display a higher expression level of the M2 cytokine IL-4ra.

Pre-treatment with CGS-21680 induced down-regulation of TNFα and IL4ra and up-regulation of IL6 gene expression in IBO-treated cells, but had no effect on gene expressions of IL1β, iNOS and IL10.

**Figure 9:**
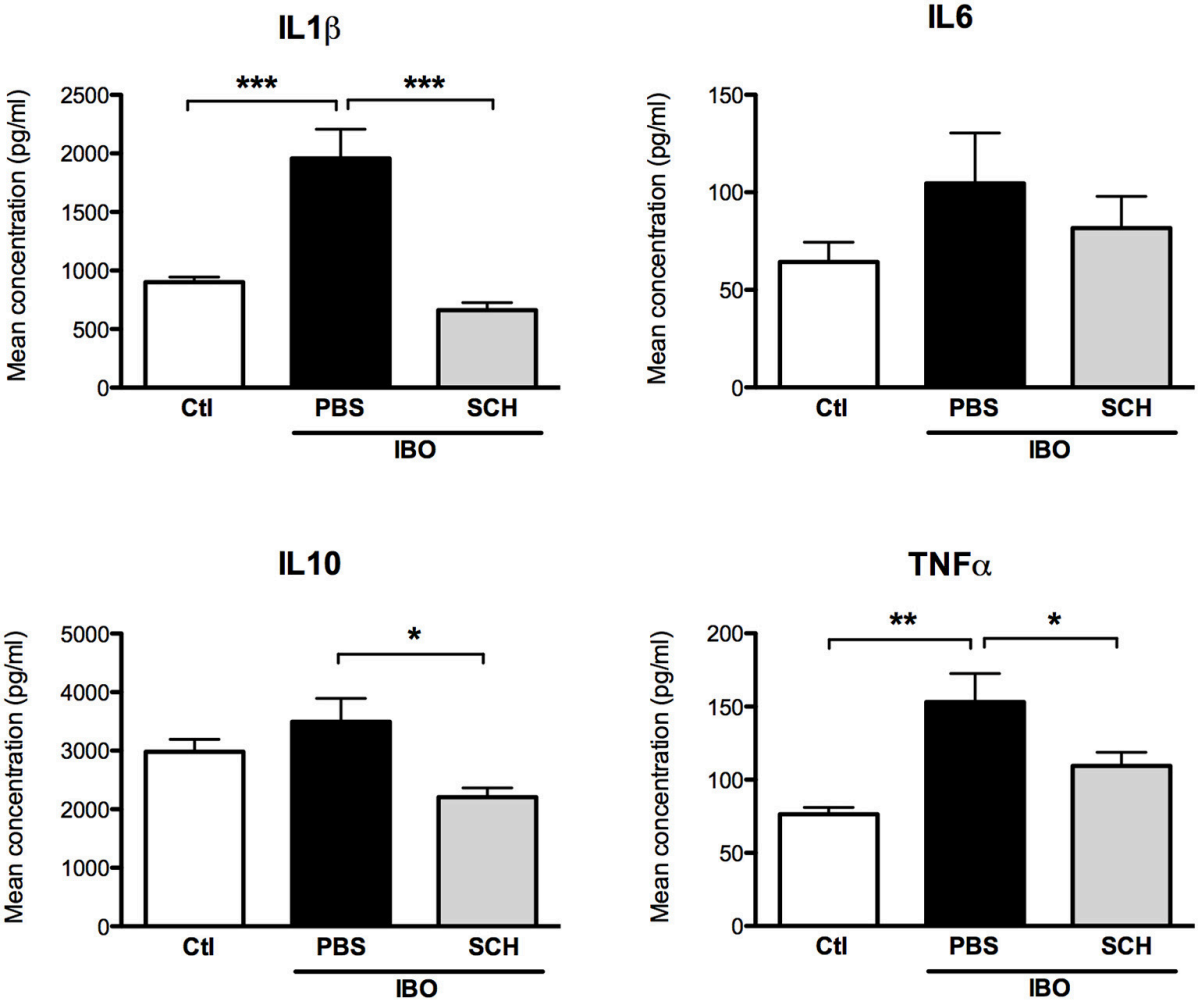
Measurement of cytokine concentrations in culture media collected 12 h after 300 μM ibotenate (IBO) exposure in microglial cells pre-treated with or without SCH. (^*^p < 0.05, ^**^p < 0.01, ^***^p < 0.001, using one-way ANOVA followed by Newman–Keuls *post-hoc* analysis).

In another set of experiments, conditioned culture media were collected after 12-h IBO exposure with or without SCH-58261 pre-treatment, and cytokine concentrations were assessed (Figure 9). While IBO induced higher IL1β and TNFα concentrations in the culture media, the A2aR antagonist SCH-58261 exposure was associated with a reduced cytokine production in response to excitotoxic challenge.

## Discussion

This study strongly suggests that adenosine and the regulation of its receptor A2aR play a role in neonatal brain inflammation and microglial activation in rat.

Abundance of literature has demonstrated both in clinical and preclinical studies that neuroinflammation is a relevant component in the pathogenesis of prematurity-related brain injury (3, 4, 9, 29–32). Interestingly, adenosine exerts a role in this context and is able to orchestrate the inflammatory response (33–35). However, its role in the neonatal brain has not yet been elucidated, even if caffeine, a non-specific adenosine antagonist, has shown an important neuroprotective role in premature infants.

The adenosine system appears to be involved in regulating inflammation in both acute (IBO) and chronic (LPD) neuro-inflammation *in vivo*. The two animal models used in the present study display alterations that occur at a developmental stage of the rat brain that corresponds to the human brain at 28–32 gestation weeks (GW) (36). This window is recognized as a period of high vulnerability for the developing brain to either excitotoxic or inflammatory insults (37). Interestingly, these effects are exerted through the modulation of microglia reactivity, that, as previously reported (23, 38), characterizes the two animal models.

In conditions of inflammation, oxidative stress, excitotoxicity or cellular necrosis, the purinergic system is the first to be involved (39). Indeed, under pathological conditions, extracellular ATP is produced by both neurons and glial cells (40) and is rapidly converted to ADP and AMP by Entpd1 and by CD73, which convert AMP to adenosine (41). Despite its very short half-life (few seconds), increased adenosine brain levels contributes to induction and modulation of neuro-inflammation (42).

To assess the adenosine implication in the LPD model, an adenosine assay on whole blood was performed. Since deliveries can span a 12-h and to avoid the stressful peak related to delivery, the blood samples were collected at P1, when LPD pups showed higher level of adenosine blood levels compared to controls. As described for human neonates (43), delivery is responsible for a physiological increase in adenosine blood levels in the newborn. Interestingly, adenosine blood levels remained higher in LPD at P4 in our study, when the effects of delivery have disappeared.

Remarkably, the increasing adenosine blood levels in LPD rats are similar to those found in premature infants (20) and suggest a pro-inflammatory condition that characterizes these babies from birth until the first month of life. Similarly to neonates, LPD pups also showed a chronic inflammatory condition, as a result of maternal malnutrition, with a detrimental effect on neurodevelopment (23). In the brain, CD73, also known as Nt5e, is considered as the principal enzyme involved in the production of extracellular adenosine (44). The mRNA expression of the two ectonucleotidases Entpd1 and CD73, responsible for the final step of ATP catabolism into adenosine, is significantly increased in LPD both at P1 and P4. These results are in agreement with the study conducted by Chen et al. who reported that the activity of the ectonucleotidases is stimulated by inflammatory conditions (45).

Adenosine elicits its physiological responses by binding to and activating one or more of the four transmembrane ARs. Solid evidence demonstrated that the four receptors are all expressed in the brain (33) and our results confirm the expression of all ARs in the rat pup cortex. Interestingly, LPD animals displayed an increased expression of A2aR and A2bR, known to exert a pro-inflammatory action (14, 46), in the pre-frontal cortex both at P1 and P4. In the LPD model, which induces fetal growth restriction, the main alteration consists in disturbance of oligodendrocytes progenitor cells (OPC) maturation conducing to a deficit of myelination, that occurred in combination with a proinflammatory state evidenced by transcriptomic analysis performed in sorted microglia (23). Our *in vitro* results revealed that microglial cells sorted from rat pups subjected to LPD have abnormal reactivity with increased Iba1 staining and smaller size, when compared to control cells. Iba1 is constitutively expressed by microglia and is involved in the actin-crosslinking associated with membrane ruffling of microglial cells, an event essential for the morphological changes from quiescent ramified microglia to activated amoeboid microglia (47). Furthermore, the reduction in cell size has been shown to be strongly correlated to microglial activation (48, 49).

Regarding the potential role of adenosine in the modulation on microglia activity, we reported an increase in A2aR transcripts and protein levels in LPD-exposed microglia cells. A2aR has an important role in the control of inflammatory events (14) by regulating microglial reactivity, changing microglial morphology (16), increasing the release of cytokines and prostaglandin E2 (15) and nitric oxide synthase activity. In addition to A2aR, CD73 has been shown to have an important role in modulation of microglia ramification and activation (51). These data are consistent with our findings and support a possible role of adenosine in the regulation of the inflammatory response following brain injury.

The results of *in vitro* studies conducted using an A2aR pharmacological approach clearly evidenced the involvement of A2aR in the regulation of inflammatory response in LPD model. These results are well supported by previous studies, which demonstrated that A2aR antagonists suppress microglia activation and IL-1β secretion in murine microglial cells exposed to an inflammatory stimulus induced by LPS (17, 18). A2aR gene disruption in mice showed a lower severity of inflammatory response and subsequent damage in different models of brain injury including ischemia/hypoxia and traumatic brain injury (50–52).

On the other hand, treatment with CGS-21680, an A2aR selective agonist, promotes the increase in M1 markers in LPD microglial cells suggesting that chronic inflammation causes microglial cells to be more susceptible to the pro-inflammatory effect of adenosine via A2a receptors.

The Ibotenate model closely mimics the pathological features observed in periventricular white matter (38). In this model of acute brain injury and inflammation, both *in vivo* and *in vitro* findings support the involvement of A2aR. However, some features of microglial activation *in vitro* appear to be different from those observed in the LPD model, as microglial cells treated by ibotenate showed no difference in cell size and Iba1 expression. In conclusion this study gives evidence of the involvement of adenosine and in particular of its receptor A2aR in the regulation of microglia in two models of perinatal brain injury associated with neuro-inflammation. The present study focused only on the relation between adenosine formation and A2aR inactivation; it remains to be explored whether the other receptors might play a role in the regulation of microglia in the same models of perinatal brain injury. Indeed, all the 4 receptors subtypes are non-specifically inactivated by caffeine. Further studies may provide a functional role for caffeine and specific antagonists of the remaining ARs in the limitation of perinatal brain injury associated with neuro-inflammation. In summary, the present study suggests that A2aR, up-regulated as consequence of inflammation, can influence the microglia phenotype, building up a potential for A2aR antagonist as a therapeutic strategy for neonatal brain damage.

## Author contributions

MC, JM, LR, and OB designed the study. MC, MZ, and JP performed experiments. MCa performed adenosine measurements. MC, JM, and OB wrote the paper. All authors revised and approved final version of the manuscript.

### Conflict of interest statement

The authors declare that the research was conducted in the absence of any commercial or financial relationships that could be construed as a potential conflict of interest.
