# Supplementary material for: Modulation of Microglial Activation by Adenosine A2a Receptor in Animal Models of Perinatal Brain Injury
Source: Front Neurol. 2018 Sep 11;9:605. doi: 10.3389/fneur.2018.00605 (PMC6141747; doi:10.3389/fneur.2018.00605)
Supplement: Supplementary file 1 [file Table_1.docx]

Supplemental Table 1: Primer sequences used in RT-qPCR experiments.

| Gene | Sequence | Gene | Sequence |
| --- | --- | --- | --- |
| Rpl13 | TCCGAAGAAGGGAGACAGTT | **Ada** | AAGTAGAGCTGCACGTCCAC |
|  | CTTCTCCTCTTCCGTGGATGG |  | TGCGTAGTCCCTCCACTGTA |
| IL1β | CACCTCTCAAGCAGAGCACAG | **Nt5e (CD73)** | TCCTGCAAGTGGGTGGAATC |
|  | GGGTTCCATGGTGAAGTCAAC |  | AGATGGGCACTCGACACTTG |
| IL6 | TCCTACCCCAACTTCCAATGCTC | **Entpd1** | TGTGCCTTCAACGGTGTCTT |
|  | TTGGATGGTCTTGGTCCTTAGCC |  | ACACTGTCGTTCGCCATCTT |
| iNOS | AAGAACTCGGGCATACCTTCAG | **Adk** | TGGAAATGAGACGGAGGCTG |
|  | GTCATGAGCAAAGGCACAGAAC |  | GGAAGAGCCTGCGTCTTTCT |
| Tnfα | AAATGGGCTCCCTCTCATCAGTTC | **Adora1** | GACCTACTTCCACACCTGCC |
|  | TCTGCTTGGTGGTTTGCTACGAC |  | CGGAGGTATCGATCCACAGC |
| IL10 | TGC CTT CAG TCA AGT GAA GAC | **Adora2a** | CCATCCCCTTCGCTATCACC |
|  | AAA CTC ATT CAT GGC CTT GTA |  | ACCAGGACAAAACAGGCGAA |
| IL4ra | ACTGGCTGGAACTGTGGTCT | **Adora2b** | GATCATCGCTGTCCTCTGGG |
|  | CATTGGTGTGGAGTGTGAGG |  | CTGGTGGCACGGTCTTTACT |
|  |  | **Adora3** | TGTCCTGTGTGCTTCTGGTC |
|  |  |  | AGCTTGACTCGCAGGTATCG |
