# Supplementary figures and images for: Modulation of Microglial Activation by Adenosine A2a Receptor in Animal Models of Perinatal Brain Injury
Source: Front Neurol. 2018 Sep 11;9:605. doi: 10.3389/fneur.2018.00605 (PMC6141747; doi:10.3389/fneur.2018.00605)

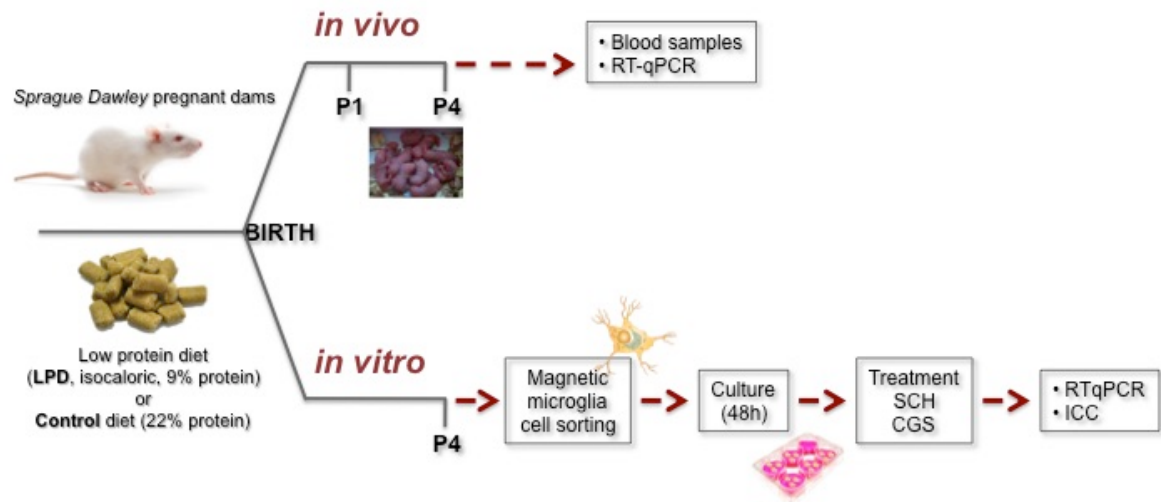

**Supplemental Figure S1:** Schematic overview of the experimental research plan using LPD model.

Supplement: Supplementary file 2 [file Data_Sheet_1.pdf]
