# Supplementary material for: Modulation of Microglial Activation by Adenosine A2a Receptor in Animal Models of Perinatal Brain Injury
Source: Front Neurol. 2018 Sep 11;9:605. doi: 10.3389/fneur.2018.00605 (PMC6141747; doi:10.3389/fneur.2018.00605)
Supplement: Supplementary file 4 [file Data_Sheet_3.pdf]

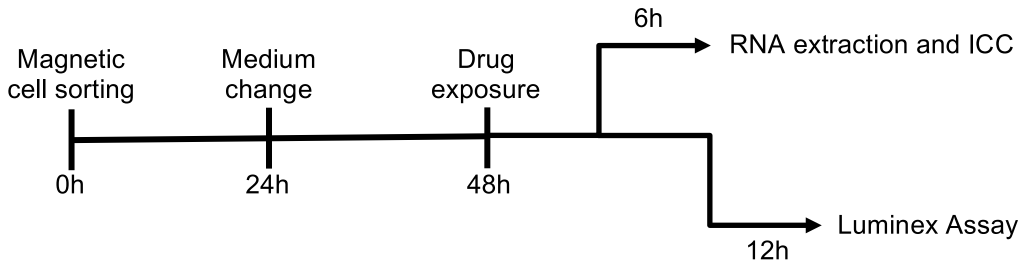

Supplemental Figure S3: Schematic representation of the experimental plan in microglial sorted cells.
