# Supplementary material for: Modulation of Microglial Activation by Adenosine A2a Receptor in Animal Models of Perinatal Brain Injury
Source: Front Neurol. 2018 Sep 11;9:605. doi: 10.3389/fneur.2018.00605 (PMC6141747; doi:10.3389/fneur.2018.00605)
Supplement: Supplementary file 5 [file Data_Sheet_4.pdf]

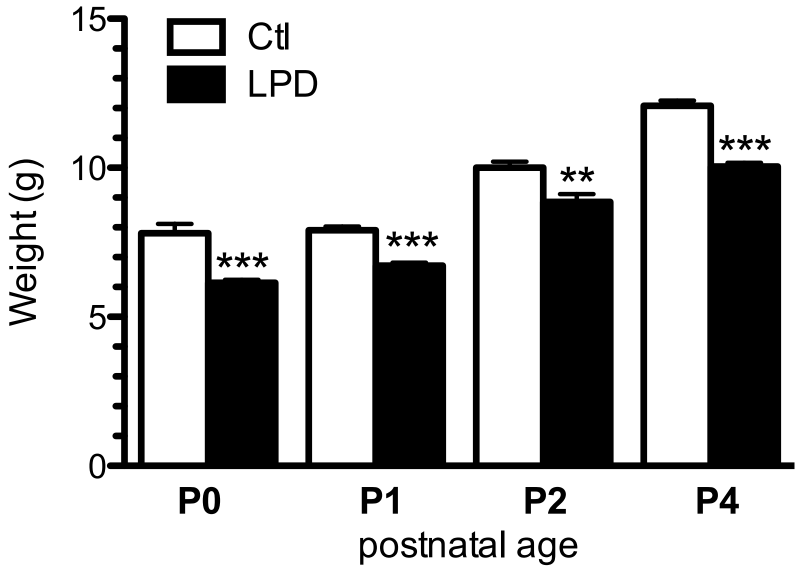

Supplemental Figure S4: Body weight of LPD pups from birth to P4 in animals exposed to prenatal Low Protein Diet (LPD) and in controls (Ctl). (**p<0.01, ***p<0.001, using unpaired Student's t test).
