# Supplementary material for: Modulation of Microglial Activation by Adenosine A2a Receptor in Animal Models of Perinatal Brain Injury
Source: Front Neurol. 2018 Sep 11;9:605. doi: 10.3389/fneur.2018.00605 (PMC6141747; doi:10.3389/fneur.2018.00605)
Supplement: Supplementary file 6 [file Data_Sheet_5.pdf]

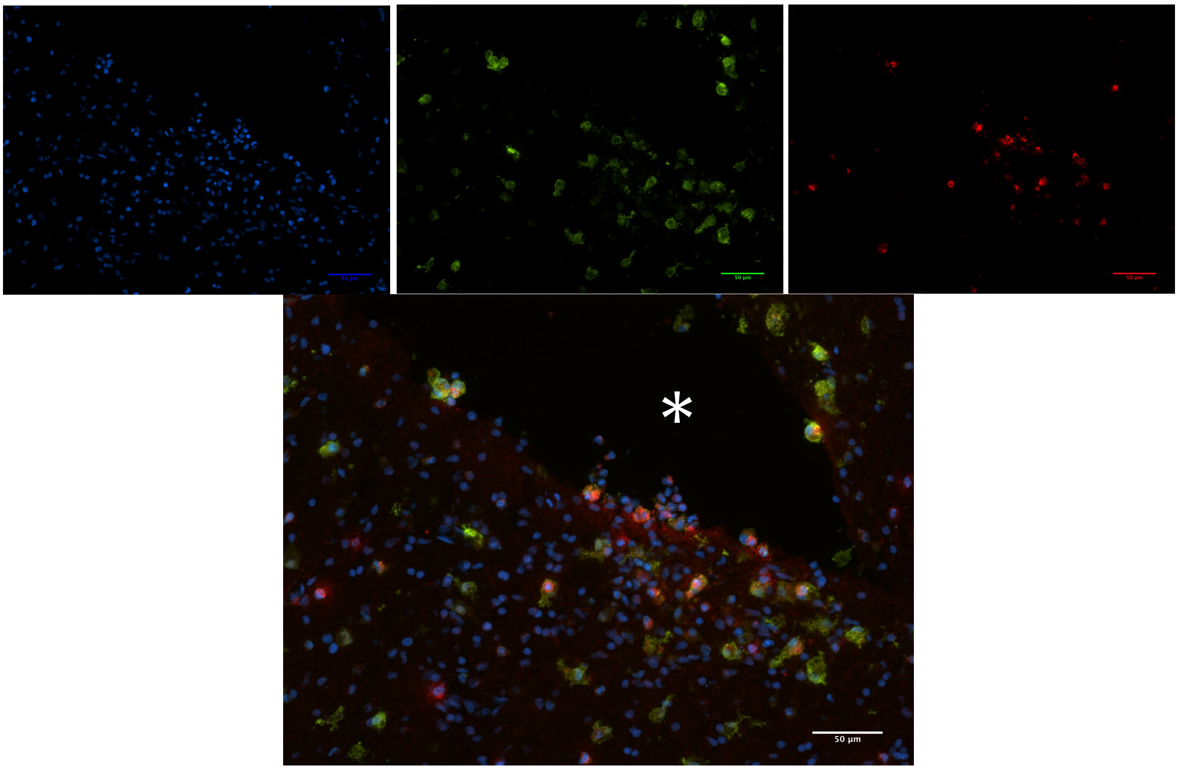

Supplemental Figure S5: Intra-cerebral Ibotenate injection increases Iba1 expression and induces reactive microglial activation at the peri-lesion site. Representative images of Iba1 staining (green) and CD73 (red) in peri-lesional white matter. Scale bars: 50µm. * white matter lesion site.
